# Supplementary material for: Localized variation in ancestral admixture identifies pilocytic astrocytoma risk loci among Latino children
Source: PLoS Genet. 2022 Sep 7;18(9):e1010388. doi: 10.1371/journal.pgen.1010388 (PMC9484652; doi:10.1371/journal.pgen.1010388)
Supplement: S1 Text — (DOCX) [file pgen.1010388.s001.docx]

| Table A: Demographic data of pediatric astrocytoma subjects | |
| --- | --- |
| Variable | Median (interquartile range) or N (%) |
| Age at diagnosis (years) | 6.0 (7.0) |
| Sex, male | 691 (51.34%) |
| Self-reported race |  |
| Non-Latino White | 799 (59.36%) |
| Latino | 547 (40.64%) |
| Histology |  |
| Pilocytic Astrocytoma | 772 (57.36%) |
| Non-pilocytic astrocytoma | 574 (42.64) |
| Primary site |  |
| Cerebellum, Not otherwise specified (NOS) | 369 (27.41%) |
| Brain stem | 187 (13.89%) |
| Cerebrum | 156 (11.59%) |
| Brain NOS | 120 (8.92%) |
| Other | 514 (38.19%) |
| Birth weight (g) | 3487.0 (659.0) |
| Gestational age (days) | 279.0 (16.0) |
| Tumor Grade |  |
| I | 167 (5.32%) |
| II | 222 (7.07%) |
| III | 14 (0.45%) |
| IV | 179 (5.70%) |
| NOS | 764 (24.33%) |

| Table B: Description of Latino non-pilocytic astrocytoma cases and controls | |
| --- | --- |
| Variable | Median (interquartile range) or N (%) |
| Sex, male | 539 (50.09%) |
| Birth weight (g) | 3405.0 (678.0) |
| Gestational age (days) | 277.0 (17.0) |
| Case status |  |
| Cases | 246 (22.86%) |
| Controls | 830 (77.14%) |
| Age at diagnosis among cases (years) | 6.0 (8.0) |
| Histologies among cases (ICD-O codes) |  |
| Diffuse astrocytoma (9400) | 125 (50.81%) |
| Anaplastic astrocytoma (9401) | 65 (26.42%) |
| Dysembryoplastic neuroepithelial tumor (9413) | 24 (9.76%) |
| Pleomorphic xanthoastrocytoma (9424) | 17 (6.91%) |
| Fibrillary astrocytoma (9420) | 11 (4.47%) |
| Gemistocytic astrocytoma (9411) | 2 (0.81%) |
| Desmoplastic infantile astrocytoma (9412) | 1 (0.41%) |
| Protoplasmic astrocytoma (9410) | 1 (0.41%) |
| Primary site among cases |  |
| Brain stem | 44 (17.89%) |
| Cerebrum | 40 (16.26%) |
| Temoral lobe | 39 (15.85%) |
| Overlapping lesion of brain | 21 (8.54%) |
| Cerebellum, NOS | 17 (6.91%) |
| Frontal lobe | 16 (6.50%) |
| Brain, NOS | 15 (6.10%) |
| Spinal cord | 13 (5.28%) |
| Parietal lobe | 13 (5.28%) |
| Others | 28 (11.38%) |
| Tumor Grade among cases |  |
| I | 13 (5.28%) |
| II | 42 (17.07%) |
| III | 5 (2.03%) |
| IV | 78 (31.71%) |
| NOS | 108 (43.90%) |

| Table C: conditional analysis in admixture mapping peaks | | | | | |
| --- | --- | --- | --- | --- | --- |
| Regional  admixture signal | Conditioned on | MAF  Lat | MAF  Eur | Estimate | Pr(>\|z\|) |
| chr6:85504599 | none |  |  | 0.465 | 4.70x10^-6^ |
| chr6:85504599 | chr6:86961711 | 0.010 | 0.025 | 0.446 | 2.09x10^-5^ |
| chr13:94441872 | none |  |  | -0.408 | 2.25x10^-5^ |
| chr13:94441872 | chr13:93952876 | 0.26 | 0.16 | -0.389 | 1.65x10^-4^ |
