# Supplementary material for: Localized variation in ancestral admixture identifies pilocytic astrocytoma risk loci among Latino children
Source: PLoS Genet. 2022 Sep 7;18(9):e1010388. doi: 10.1371/journal.pgen.1010388 (PMC9484652; doi:10.1371/journal.pgen.1010388)
Supplement: Attachment — Submitted filename: Review of Li et al_2022.docx [file pgen.1010388.s002.docx]

Review of Li et al, 2022

This paper describes the results of a Latino admixture-based scan for genetic regions that harbor variants related to the occurrence of pilocytic astrocytoma and which may partially explain the higher risk of this tumor in children of European ancestry compared to other ancestries, and in particular lower risk among Latinos. While benign, slow-growing, and generally curable by resection, due to the sensitive intra-cranial locations such as the optic nerve pathway, and the relatively high frequency of occurrence (it is the most common pediatric brain tumor), studies of genetic (and non-genetic) susceptibility is certainly welcome and warranted. The biggest weakness of this study is the lack of a replication either of the admixture signal or the SNPs with the smallest p-values within the region of interest. The authors should comment on this weakness

Overall, I find that this study is well designed and executed, I have only a few comments/questions/suggestions.

1. Admixture mapping was originally described as a case-only analysis looking for regions where cases were significantly enriched with a specific local ancestry, compared to other regions in the genome. The case-only analysis can be more powerful than case-control analysis if basic assumptions are met, specifically that there are no other selective forces that would enrich local ancestry in both the controls and cases. Here do the significant or close to significant regions show a rise in local European ancestry among the controls as well as cases?
2. Page 4, Introduction, second paragraph. I find the reference to Neurofibromatosis Type 1 to be rather cryptic, what is really being stated here?
3. Page 7, sixth line from bottom. Filtering on HWE is somewhat problematic in an incompletely admixed population like (California) Latinos
4. Figure 3, Shouldn’t the description of plot 3B (in the caption of 3B) read

**3B.** boxplot showing distribution of estimated global Amerindian ancestry proportions, and ~~contrary to~~ in correspondence with 3A, cases have a lower median proportion of ~~European~~ Amerindian ancestry proportion than controls

5. Table 1. Please give risk allele frequency in this table by population (needed to compute power to replicate in other similar studies)
6. Page 17, second sentence of discussion “Specifically, each 5% increase in European

ancestry proportion associated with a 1.051-fold increase in odds of PA among Latinos (95% CI:

1.014-1.091)” This finding should be first given in the Results section not in Discussion

7. Same with the sentence page 18 “1 additional copy of the European ancestral haplotype was associated with 1.59-fold increased odds of PA.”
8. It is confusing to use the term fine-mapping in the statement that the “ (smallest P-value from fine-mapping at chr6:85504599; (was) P=4.70x10-6)”. This sounds like a statement about one of the SNPs considered when fine-mapping the admixture scan signal, rather than the maximal signal from the admixture scan for chromosome 6.
9. The p-value for global significance of 7.75 x 10-6, from STEAM should be easier to find and to compare to the observed smallest p-value of 4.7x10-6, the STEAM result now is only given in the figure caption for fig. 4.
