# Supplementary material for: Localized variation in ancestral admixture identifies pilocytic astrocytoma risk loci among Latino children
Source: PLoS Genet. 2022 Sep 7;18(9):e1010388. doi: 10.1371/journal.pgen.1010388 (PMC9484652; doi:10.1371/journal.pgen.1010388)
Supplement: Attachment — Submitted filename: admixture_mapping_response.docx [file pgen.1010388.s003.docx]

Dear Editor,

We thank you for the careful review of our manuscript, “Localized variation in ancestral admixture identifies pilocytic astrocytoma risk loci among Latino children.” The reviewer comments were insightful and aided in a revision of the manuscript which we believe is much improved. Below we write a point-by-point response to the reviewer comments indicating where we have changed the presentation in the manuscript. We hope that these revisions are deemed sufficient and we look forward to further evaluations.

We also submit an image and caption for consideration for your “striking image” request.

Sincerely,

Joseph Wiemels for Shaobo Li and all the authors

Reviewer 1

- Lack of a replication either of the admixture signal or the SNPs with the smallest p-values within the region of interest. The authors should comment on this weakness

***Response:*** We appreciate this comment, and we have included the following text in the discussion section to address lack of replication:

“While the presence of one and up to three regions were identified in this admixture analysis, we acknowledge the lack of a replication in an independent dataset as a weakness to the current study. We would therefore encourage replication in future cohorts of pilocytic tumors particularly those derived from EUR populations.”

- Admixture mapping was originally described as a case-only analysis looking for regions where cases were significantly enriched with a specific local ancestry, compared to other regions in the genome. The case-only analysis can be more powerful than case-control analysis if basic assumptions are met, specifically that there are no other selective forces that would enrich local ancestry in both the controls and cases. Here do the significant or close to significant regions show a rise in local European ancestry among the controls as well as cases?

***Response:*** Thank you very much for this comment. We tested the level of European ancestry by case-only and control-only status (separately) around significant peak in Chr 6 as discussed in Figure 3 of Winkler CA et al [1], and then graphed them together.

Zooming in to the Chr 6 peak, we can see an increase in European ancestry proportions in cases comparing to controls at our position of interest (arrow). although comparing to regions around this peak, European ancestry proportions were not significantly higher, either in cases or controls.

The difference in case and control EUR ancestral status over the broad region indicated that this region may be subject to selective forces as the reviewer suggested as a possibility, suppressing EUR ancestry over the population while not removing a EUR-specific risk factor(s). Another possibility is that a rare, ungenotyped and unimputed variant(s) associated with EUR status, or more than one, is contained within this region and linked to the genotyped variants.

*[1] Winkler CA, Nelson GW, Smith MW. Admixture mapping comes of age. Annu Rev Genomics Hum Genet. 2010;11:65-89. doi: 10.1146/annurev-genom-082509-141523. PMID: 20594047; PMCID: PMC7454031.*

- Page 4, Introduction, second paragraph. I find the reference to Neurofibromatosis Type 1 to be rather cryptic, what is really being stated here?

***Response:*** Thank you for pointing this out. We discussed NF1 here to show this is a rare situation where we understand a strong predisposition risk factor for PA, given that NF1 patients have a higher chance of contracting PA, via mutations of the NF1 gene. To make our point more clear, we have adjusted the text to the following:

“… heritable genetic contributions impacting risk of PA remain largely unidentified, other than in the context of Neurofibromatosis Type I, where it was shown Neurofibromatosis Type I patients have a higher chance of contracting optic pathway PA, most likely due to *NF1* germline mutations.”

- Page 7, sixth line from bottom. Filtering on HWE is somewhat problematic in an incompletely admixed population like (California) Latinos

***Response:*** This is a valid concern. Indeed, in a recently admixed population, HWE cut-off will be an issue since there is not enough generations to allow equilibrium to occur for alleles with different ancestral-associated allele frequencies. We acknowledge this as a potential problem.

To address this, we adopted a less stringent cut-off, using 10^-4^, instead of 10^-5 or -6^; additionally we did not filter on cases since risk variants can disrupt HWE, only controls. Additionally, for SNPs that were filtered out from the HWE cut-off over the whole population, most should be imputed back during the whole genome imputation step for each individual, which should further alleviate this issue. We have added a reference [1] and justification to this manuscript, to better address this issue.

*[1] Brief Funct Genomics: 2016 Jul;15(4):298-304. doi: 10.1093/bfgp/elv037. Epub 2015 Oct 5.*

*Quality control, imputation and analysis of genome-wide genotyping data from the Illumina HumanCoreExome microarray. Jonathan R I Coleman, Jack Euesden, Hamel Patel, Amos A Folarin, Stephen Newhouse, Gerome Breen*

- Figure 3, Shouldn’t the description of plot 3B (in the caption of 3B) read

**3B.** boxplot showing distribution of estimated global Amerindian ancestry proportions, and ~~contrary to~~ in correspondence with 3A, cases have a lower median proportion of ~~European~~ Amerindian ancestry proportion than controls

***Response:*** We have adjusted our text accordingly. We really appreciate you pointing this out for us.

- Table 1. Please give risk allele frequency in this table by population (needed to compute power to replicate in other similar studies)

***Response:*** We have updated our Table 1 to include risk allele frequency in each population.

- Page 17, second sentence of discussion “Specifically, each 5% increase in European

ancestry proportion associated with a 1.051-fold increase in odds of PA among Latinos (95% CI: 1.014-1.091)” This finding should be first given in the Results section not in Discussion

- Same with the sentence page 18 “1 additional copy of the European ancestral haplotype was associated with 1.59-fold increased odds of PA.”

***Response:*** Thank you very much for the suggestion, we have moved this text to the results section and modified the discussion part accordingly.

- It is confusing to use the term fine-mapping in the statement that the “ (smallest P-value from fine-mapping at chr6:85504599; (was) P=4.70x10-6)”. This sounds like a statement about one of the SNPs considered when fine-mapping the admixture scan signal, rather than the maximal signal from the admixture scan for chromosome 6.

***Response:*** Thanks for this suggestion. To make it clearer, we have modified the text to: “smallest P-value from **admixture mapping** at chr6:85504599; P = 4.70x10^-6^”, and moved it into the results section per last comment.

- The p-value for global significance of 7.75 x 10-6, from STEAM should be easier to find and to compare to the observed smallest p-value of 4.7x10-6, the STEAM result now is only given in the figure caption for fig. 4.

***Response:*** Thank you and we really appreciate this suggestion. We have now added text in the results section to emphasis the significance threshold: “Genome-wide significance level was determined by STEAM to be 7.75 x 10^-6^ as shown in Figure 4A”.

Reviewer 2

- The current gold standard program to infer global ancestry is RFMix. Please justify the use of ADMIXTURE, since RFMix was used to infer local ancestry.

***Response:*** Thank you for this comment. We chose to use ADMIXTURE to estimate global ancestry proportion as it has been part of our lab’s pipeline and has proven to produce fast and accurate results, and highly specific for estimating global ancestry. An example of previous publication includes [1]. Admittedly, it has been reported that RFMix outperforms ADMIXUTURE in general, however, to date, there is not yet multi-cohort, large-scale formal comparisons of different ancestry estimation tools to really establish any software as gold standard. Furthermore, it has also been shown that the results of these two software are highly correlated [2] [3] [4].

We also checked the consistency between global ancestry proportions calculated by ADMIXTURE and RFMix (Comparing global African, Amerindian and European ancestries and there is a high correlation between their results (R^2^ ≥ 0.98, data not shown).

We also repeated comparison of global ancestries using RFMix results instead, and the results remain significant. More specifically, in Latino populations, PA cases have a significantly higher proportion of European ancestry (P value = 0.004218), and a lower Amerindian ancestry proportion (P value = 0.001471). Box-plots similar to 3A and 3B are shown below. We have included a mention of this in the revised manuscript, p 11: “Essentially identical results were calculated when RFMix output was used instead of ADMIXTURE for ancestry calculations.”

RFMix output used for ancestry comparison as in Fig 3, manuscript

*[1] Zhang C, Ostrom QT, Hansen HM, Gonzalez-Maya J, Hu D, Ziv E, Morimoto L, de Smith AJ, Muskens IS, Kline CN, Vaksman Z, Hakonarson H, Diskin SJ, Kruchko C, Barnholtz-Sloan JS, Ramaswamy V, Ali-Osman F, Bondy ML, Taylor MD, Metayer C, Wiemels JL, Walsh KM. European genetic ancestry associated with risk of childhood ependymoma. Neuro Oncol. 2020 Nov 26;22(11):1637-1646. doi: 10.1093/neuonc/noaa130. PMID: 32607579; PMCID: PMC7846152.*

*[2] Moreno-Estrada A, Gravel S, Zakharia F, McCauley JL, Byrnes JK, Gignoux CR, Ortiz-Tello PA, Martínez RJ, Hedges DJ, Morris RW, Eng C, Sandoval K, Acevedo-Acevedo S, Norman PJ, Layrisse Z, Parham P, Martínez-Cruzado JC, Burchard EG, Cuccaro ML, Martin ER, Bustamante CD. Reconstructing the population genetic history of the Caribbean. PLoS Genet. 2013 Nov;9(11):e1003925. doi: 10.1371/journal.pgen.1003925. Epub 2013 Nov 14. PMID: 24244192; PMCID: PMC3828151.*

*[3] Martin AR, Gignoux CR, Walters RK, Wojcik GL, Neale BM, Gravel S, Daly MJ, Bustamante CD, Kenny EE. Human Demographic History Impacts Genetic Risk Prediction across Diverse Populations. Am J Hum Genet. 2017 Apr 6;100(4):635-649. doi: 10.1016/j.ajhg.2017.03.004. Epub 2017 Mar 30. Erratum in: Am J Hum Genet. 2020 Oct 1;107(4):788-789. PMID: 28366442; PMCID: PMC5384097.*

*[4] Fortes-Lima C, Gessain A, Ruiz-Linares A, Bortolini MC, Migot-Nabias F, Bellis G, Moreno-Mayar JV, Restrepo BN, Rojas W, Avendaño-Tamayo E, Bedoya G, Orlando L, Salas A, Helgason A, Gilbert MTP, Sikora M, Schroeder H, Dugoujon JM. Genome-wide Ancestry and Demographic History of African-Descendant Maroon Communities from French Guiana and Suriname. Am J Hum Genet. 2017 Nov 2;101(5):725-736. doi: 10.1016/j.ajhg.2017.09.021. PMID: 29100086; PMCID: PMC5673667.*

- After merging with the reference population, what was the SNP density?

***Response:*** Thank you very much for this question. There is a high overlap between our query panel and reference panel.

In terms of specific numbers, in the original data, a total of 764,144 SNPs were available before merging. 719,803 SNPs (94.20%) were also in the reference panel.

We also did whole genome imputation before calculating regional ancestry using RFMix, and for both reference and query populations, a total of 73,159,508 SNPs were imputed, and this equates to 2.3 SNPs per 1000 bp on average throughout the genome.

- Although I am sure the appropriate IRB approvals were obtained from the California Department of Public Health Institutional Review Board, none of the approval numbers are listed in the manuscript.

***Response:*** Thank you for this comment. We have included the following text in the method section, including our IRB project number:

“The State of California Committee for the Protection of Human Subjects (CPHS), as well as the University of Southern California and University of California, Berkeley institutional review boards approved this study. The CPHS IRB Project number is 15-05-2005.”

- Please show the individual values of the ancestry proportions in the box plots in Figure 3.

***Response:*** Thank you for this comment. We have now updated our Figure 3 and all box plots now also include individual observations.

- Please remove references to “race” in the manuscript. To quote, “Race is a social construct” as indicated by the American Society of Human Genetics. ([https://www.cell.com/ajhg/fulltext/S0002-9297(18)30363-X](https://urldefense.com/v3/__https:/www.cell.com/ajhg/fulltext/S0002-9297(18)30363-X__;!!LIr3w8kk_Xxm!oR2__8-4dPHAxxuLtqx6Z093rY5yQ58FqZ7vZtg8WyEJBUtVcKX5ZZ2WwvFFzTd8oQDqCadTt8kvuOc-$) ) Rather use ethnicity or ancestry, as you have done throughout most of the text.

***Response:*** Thank you for this suggestion. We used “race” three times in the text and we have changed all of them to “ethnicity” or “ancestry” to be more accurate.
